# Active touch in tactile perceptual discrimination: brain activity and behavioral responses to surface differences

**DOI:** 10.1007/s00221-025-07034-7

**Published:** 2025-03-06

**Authors:** Håkan Fischer, Elizabeth S. Collier, Amirhossein Manzouri, Kathryn L. Harris, Lisa Skedung, Mark W. Rutland

**Affiliations:** 1 Department of Psychology, Stockholm University, Albanovägen 12, Stockholm, 114 19 Sweden; 2 Stockholm University Brain Imaging Centre (SUBIC), Stockholm, Sweden; 3 Aging Research Center, Karolinska Institutet, Stockholm, Sweden; 4 Unit Perception & Design, Division Bioeconomy & Health, RISE Research Institutes of Sweden, Stockholm, Sweden; 5 Department of Chemistry, KTH Royal Technical Institute, Stockholm, Sweden; 6 Department of Clinical Neuroscience, Karolinska Institutet, Stockholm, Sweden; 7 Division of Society & Health, Department of Health, Medicine and Caring Sciences, Linköping University, Linköping, Sweden

**Keywords:** Active touch, Brain, fMRI, Tribology

## Abstract

This study investigates the neural and behavioral mechanisms of tactile perceptual discrimination using fMRI and a set of wrinkled surface stimuli with varying textures. Fifteen female participants were tasked with distinguishing between different surfaces by touch alone. Behavioral results demonstrated variable discriminability across conditions, reflecting the tactile sensitivity of human fingertips. Neural analysis showed varied brain activations tied to the task’s difficulty. In the easiest least fine-grained discrimination condition, widespread activations were observed across sensory and integration regions. As task difficulty increased, stronger parietal and frontal lobe involvement reflected higher cognitive demands. In the hardest most fine-grained discrimination condition, activation concentrated in the right frontal lobe, indicating reliance on executive functions. These results highlight the brain’s intricate role in processing sensory information during tactile discrimination tasks of varying difficulty. As task difficulty increases, the brain adapts by engaging additional neural resources to meet higher cognitive demands. This research advances our understanding of the psychophysical and neural bases of tactile discrimination acuity, with practical implications for designing materials that enhance tactile feedback.

**Supplementary Information:**

The online version contains supplementary material available at 10.1007/s00221-025-07034-7.

## Introduction

The human sense of touch has been a subject of scientific inquiry for over a century, with foundational work by David Katz in the early 20th century laying the groundwork for our understanding of texture perception through touch. Katz’s seminal work, “The World of Touch,” (1989) first published in 1925 (Katz 1925), highlighted the unique ability of the tactile system to perceive surface textures, differentiating between smoothness, roughness, and other tactile qualities. This historical perspective provides essential context for contemporary studies, which have significantly advanced our understanding of how the skin, particularly the fingertips, interacts with various surfaces to decode texture, shape, and material.

Tactile perception is a broad field and the psychotribology (Skedung et al. 2018a) of active dynamic touch treated in this work is only one aspect. Thus we exclude passive touch (Ackerley et al. 2012), affective touch (McGlone et al. 2014; Olausson et al. 2002), kinaesthetic /proprioceptive aspects (Collier and Lawson 2016) of touch 3D spatial perception (Bergmann Tiest et al. 2012) (and the so called intra-active touch– where an object is moved over the body (Bolanowski et al. 1999). Touch is not merely a passive experience but involves an active and sophisticated decoding process. When our fingers encounter an object dynamically, they engage in a complex orchestration of mechanical interactions. These depend on the surface topography and other properties, which include frictional forces, and vibrations, which the skin’s mechanoreceptors translate into a rich array of tactile sensations (Skedung et al. 2013). Contemporary research has demonstrated that the human fingertip exhibits extraordinary tactile acuity, capable of discerning minute differences in surface properties, a sensitivity that has been corroborated in various psychophysical and neurophysiological studies (Carpenter et al. 2018; Gueorguiev et al. 2016; Kuroki et al. 2017; Skedung et al. 2018b).

This intricate relationship between surface properties and tactile perception is crucial for understanding how humans navigate their environment. Investigations into the neural mechanisms of tactile discrimination have revealed that four primary mechanoreceptors in the glabrous skin of the hand—Meissner corpuscles, Merkel discs, Ruffini endings, and Pacinian corpuscles—play distinct roles in transducing different tactile stimuli, the roles of which are described in detail elsewhere. Briefly Meissner and Pacinian corpuscles are both fast adapting receptors sensitive to vibrations of different frequencies, the latter is also sensitive to pressure. Ruffini endings and Merkel discs are slow adapting receptors implicated in skin stretching and deformation. These mechanoreceptors (Johnson 2001; Fleming and Luo 2013) generate action potentials that are processed by the central nervous system, forming the basis of tactile perception. Recent evidence suggests that there may be a hierarchy in the deployment of mechanoreceptors for tactile acuity, with vibrotactile pathways playing a primary role, while slowly adapting (SA) receptors contribute by encoding spatial properties of stimuli, which can support performance in tasks with increasing complexity (Skedung et al. 2018b). Recent advancements in brain imaging, particularly functional magnetic resonance imaging (fMRI), have offered significant insights into how the brain decodes these tactile signals, revealing that the somatosensory cortex (S1) is highly involved in texture discrimination (Lederman et al. 2001; Simões-Franklin et al. 2011; Henderson et al. 2023).

Building on this foundation, modern research has focused on identifying the specific neural circuits involved in texture perception. For example, different populations of neurons within S1 are activated by distinct features of tactile stimuli, such as roughness or fine patterns, suggesting a highly organized tactile map in the brain. Furthermore, the secondary somatosensory cortex (S2) has been implicated in integrating tactile information with other sensory modalities, facilitating a more comprehensive perception of texture (Harris et al. 2001; Hua-Chun et al. 2016). These findings underscore that texture discrimination is not purely tactile but also involves complex neural processing and multisensory integration, allowing for nuanced interpretations of tactile experiences.

The present study builds upon this rich history by employing an active touch paradigm, where participants actively explore surfaces, as this better reflects real-world tactile interactions. Active touch, which engages both the sensory and motor systems, has been shown to elicit greater activation in contralateral S1 regions compared to passive touch, highlighting the importance of active exploration in tactile perception (Simões-Franklin et al. 2011). This approach offers a more ecologically valid means of investigating the behavioral and neural correlates of tactile discrimination which is the aim of this functional-magnetic- resonance-imaging (fMRI) study. The aim of the current parametrically designed study is to investigate how the brain processes increasing levels of difficulty in tactile perceptual decisions. Task difficulty likely reflects a combination of factors, including how stimuli are encoded by different mechanoreceptors, the variability in self-generated exploratory movements (which can vary both within and between trials, as well as across different surfaces), and the cognitive demands associated with comparing surface structures, particularly in terms of working memory and attentional resource allocation. These factors may interact in complex ways, influencing both behavioral performance and neural activation patterns. We hypothesize that finer (i.e., more complex and difficult) discriminations, compared to less fine-grained and easier discriminations, are associated with changes in both perceptual and cognitive processes, reflecting differences in their neural underpinnings.

## Methods

The study was conducted in compliance with the Declaration of Helsinki (2013) and personal data was handled in compliance with the General Data Protection Regulation (EU) 2016/679 (GDPR). All participants provided written informed consent before participation. Ethical approval was obtained from the Swedish Research Ethics Authority, application number 2019–04584.

### Participants

Fifteen right-handed (self-reported) female participants (mean age = 23.33 years; SD = 3.18 years) were recruited through advertising on an online research recruitment platform. Participants were compensated with a gift voucher worth 500 SEK.

### Stimuli and apparatus

A subset of the wrinkled surface stimuli, also used in Skedung et al. (2013) and Arvidsson et al. (2017), were used as stimuli: three surfaces with regular, uniform, sinusoidal patterns of wavelengths 20 μm (S20), 60 μm (S60), and 100 μm (S100), as well as a smooth untextured surface (S0). These surfaces created using the principle of surface wrinkling where the fabrication method has been described elsewhere (e.g. Skedung et al. 2013). In short, a cured PDMS substrate (polydimethoxysiloxane, Sylgard 184 Dow Corning, USA) is mounted on a strain stage, stretched, and in the stretched state it is being exposed to ultraviolet ozone irradiation. This generates a stiffer film on the top surface, and when the strain is released a surface wrinkles spontaneously form perpendicular to the direction of the strain as a result of the mismatch in elastic modulus between the stiffer top layer and softer substrate. By varying the exposure time and compressive strains, wrinkles of specific wavelengths can be produced. The pattern from the PDMS substrate is then replicated into a durable, cleanable surface replica using a UC-curable adhesive (Norland Optical Adhesive 81, Norland Products Inc., Cranbury, USA). Replicated untreated PDMS-specimens with no systematic sinusoidal texture is used to get the smooth untextured surface (S0) in the same durable material. The generated wavelengths were quantified using a stylus profilometer (DektakXT Profiler, Nano GmbH, Germany). Line scans of 1.1 mm as well as area images of 1 × 1 cm acquired from 250 lines obtained with a stylus (radius 2 μm) were obtained by moving the stylus tip across the wrinkles. The wavelength was obtained from the stylus analysis and the parameter PSm - the average peak spacing. The force of the stylus on the surface was set to 3 mg. The same wavelengths are measured (within error) on surfaces that have been used in testing, confirming that the wrinkles are robust towards repeated touching. See supplementary materials Fig. 2 for examples.

### Procedure

Timed instructions, written in E-Prime™, were delivered to the experimenter via headphones and to the participant via a screen which was fully visible inside the scanner due to the strategic placement of a mirror over the eyes. A purpose-built plastic table with adjustable height and tilt was placed over the participant’s waist. Participants rested the wrist of their dominant hand against the table with the fingers in the air until a visual cue appeared on a screen informing them to place their finger down and touch the surface. The experimenter who was in the scanner room with the participant, presenting the stimuli, received audial instructions (in headphones) about which surfaces to present. At the same time the visual instructions to either “touch” or “respond” were delivered to the participants. There were four pair types (S0-S100, S20-S100, S60-S100 and S100-S100) where the method of constant stimuli was employed, making S100 the comparator. The S100-S100 pair is the “no difference” task. The other three pair types are referred to as the “least difficult tactile discrimination task” or “Easiest” (S0-S100), the “medium difficult” or “Medium” (S20-S100), and the “most difficult task” or “Hardest” (S60-S100), based on the results of Skedung et al. (2020). See supplementary materials Fig. 1 for an example of a design matrix from one of the first-level contrasts.

On a given trial, a surface was presented for 3 s, followed by a 3 s gap after which the second surface was presented also for 3 s. After another ~ 3 s gap, a length which was jittered by 1, 1.5–2 s, participants were given 4 s to respond, using a button box which they held in their left hand, whether the surfaces felt the same (right button) or different (left button). There were four blocks of 32 experimental trials, with 8 presentations of each pair type in each block (total number of trials per participant = 128). The duration of each block was 9,5 min. Between blocks, participants were given a 2-minute rest. After two blocks, a T1 structural scan was performed (lasting 5 min 30 s), and then the final two blocks were completed. The comparator (S100) was presented first on half of the trials, and second in the other half. The total duration for this tactile task in the scanner was 55 min.

### Behaviour data analysis

Our data analyses follow established methods for calculating d-prime values at the individual level, including criteria for handling 100% and 0% hit or false alarm rates. The statistical modelling is based on these individual d-prime values.

### Brain data acquisition

Participants were scanned at Stockholm University Brain Imaging Centre (SUBIC) in a 3T whole-body magnetic resonance imaging (MRI) scanner (Prisma; Siemens, Erlangen, Germany) with a 64-element phased-array head coil. To obtain functional images, we employed a multiband echo-planar imaging (EPI) sequence that collected multiple slices simultaneously, reducing the repetition time (TR) per volume [14]. Specifically, the following parameters were used: gradient-echo EPI, TR = 1640ms, multiband factor = 2, echo time (TE) = 30 ms, flip angle = 70°, 54 axial slices of 2 mm thickness with a 25% slice gap, field-of-view = 192 × 192 mm^2^, and in-plane resolution = 2.0 × 2.0 mm^2^. Further, T1-weighted high-resolution anatomical images were acquired for each participant using magnetization-prepared rapid acquisition gradient echo (MP-RAGE) sequences (TR = 2300 ms, TE = 2.98 ms, flip angle = 9°, and voxel size = 1 × 1 × 1 mm^3^).

Functional images were preprocessed and analyzed using a General Linear Model analysis approach and the Statistical Parametric Mapping software (SPM12, http://www.fil.ion.ucl.ac.uk/spm/). The functional images were first realigned and then a slice timing correction was applied. Then the fMRI images were co-registered to structural T1-weighted images which were segmented into gray matter, white matter, and cerebrospinal fluid using the improved unified segmentation algorithm that employs an extended set of tissue probability maps (Ashburner and Friston 2005). Finally, the data were spatially normalized to the Montreal Neurological Institute (MNI) space with a 2 mm isotropic voxel size using the deformation fields derived from the segmentation of the T1-weighted images and smoothed with a 6 mm full-width-half-maximum (FWHM) isotropic Gaussian kernel.

### Brain data analysis

We included data from all four runs in a general linear model (GLM) for each subject, except for one subject who had only three runs. Initially, we modeled seven different trial types separately (S0 - S100; S100 - S0; S20 - S100; S100 - S20; S60 - S100; S100 - S60; S100 - S100) for each run to investigate potential order effects. However, since no significant order effects were found, we proceeded with a final model focusing on the four main trial conditions (see below).

In the final model, we defined four variables to represent the decision period for the four main trial conditions (S0– S100, S20– S100, S60– S100, and S100– S100), irrespective of the order in which the surfaces were presented. The decision period was assumed to start upon touching the second surface and continue until the response. For each run, the model also included four variables to account for the first surface touch (S0, S20, S60, or S100), with a fixed duration of 3 s, and an additional variable to model the response period for all trials, starting from the presentation of the response cue up to the key response. Finally, six rigid-body realignment parameters were included to account for potential motion effects.

The GLM was fitted using SPM12, incorporating a canonical hemodynamic response function and a 180-second high-pass filter. Three contrasts were calculated at the first level, including pairwise comparisons between the four main trial conditions (e.g., S0-S100 vs. S100-S100, S20-S100 vs. S100-S100, S60-S100 vs. S100-S100). These contrast images per subject were then used for the group analysis.

At the second level, a one-sample t-test was conducted to identify significant activations for each contrast. The results are reported at the cluster level, using an initial threshold of *p* < 0.001, with a family-wise error (FWE) correction applied, and significance set at *p* < 0.05.

## Results

### Tactile acuity

**Fig. 1:**
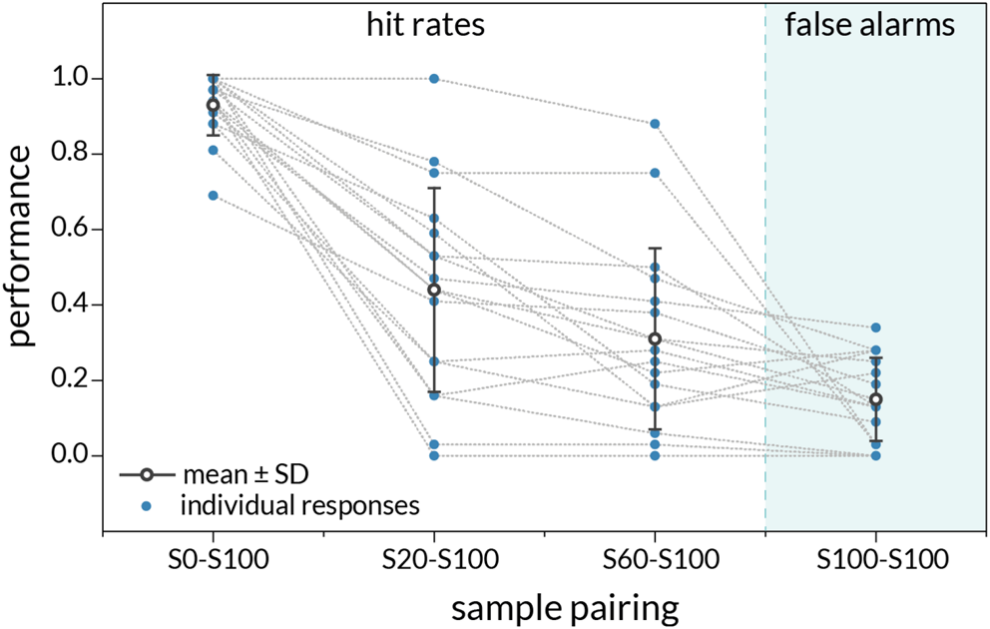
Tactile discrimination performance mean and individual hit rates for S0-S100, S20-S100, and S60-S100 conditions, as well as mean and individual false alarm rates for the S100-S100 condition. Good performance is indicated by high hit rates and low false alarm rates

The mean hit rates (p[“different”| Different]) for the easiest (S0-S100), medium difficulty (S20-S100), and hardest (S60-S100) conditions and values for each participant are shown in Fig. 1, alongside the mean and individual false alarm rates (p[“different”| Same]).

**Fig. 2:**
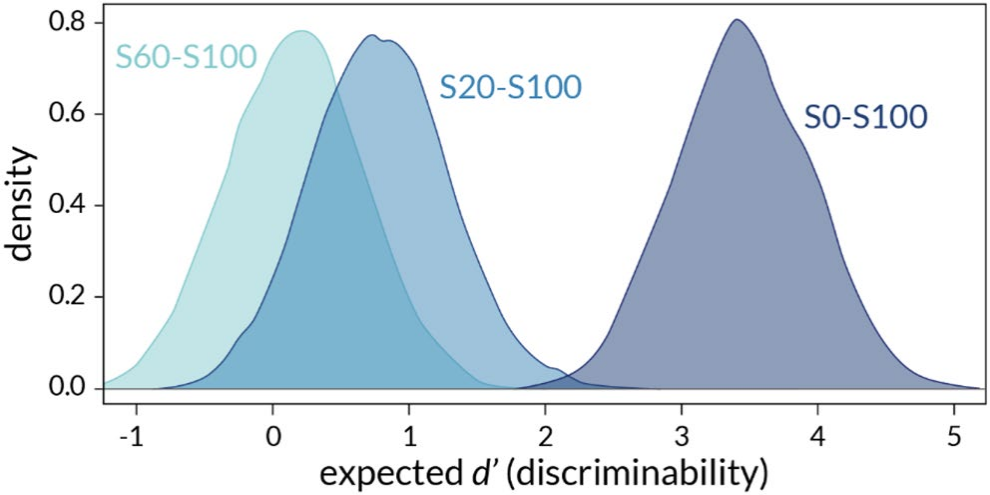
Posterior distributions of d’ for each tactile condition

Based on these hits and false alarms, the sensitivity parameter *d’* was estimated for each participant in each condition, using the *psyphy::dprime.SD* in R (version 4.1.1). For the calculation of *d’*, hit rates and false alarms of 1 or 0 were replaced with [1 − (2N)^−1^] and [2N]^−1^ respectively, where N is the number of trials that surface pair were presented. The data were analysed using a multilevel normal regression model accounting for data nesting across participants, since a within-participants design was used. The model was estimated with Bayesian inference (Bendtsen 2018; McElreath 2018) using Hamiltonian Monte Carlo (*rethinking::ulam* in R; normal priors for all coefficients, µ = 1; σ = 2; exponential priors for errors). Figure 2 shows the posterior distributions. Posterior means were used as an estimate for the most probable *d’* value obtained in that condition, and the probability of detecting *d’* values greater than 1, 2, and 4 were also calculated, see Table 1. It can be seen that discriminability is reliably higher and that there is a markedly greater probability of observing higher *d’* values for S0-S100 than in the other two conditions. Indeed, complementary repeated measures ANOVA showed that *d’* differed significantly across the conditions, *F*(2, 30) = 61.96, *p* < 0.001, η_p_^2^ = 0.81. Bonferroni-adjusted pairwise comparisons showed that *d’* was significantly higher for the S0-S100 condition than either of the other conditions, but no significant difference was detected between the S20-S100 and S60-S100 conditions.

**Table 1:** The most probable value of d’ (mean of posterior distribution) for each condition according to bayesian inference and their 95% compatibility intervals (CoIs), as well as the posterior probability of obtaining d’ values above 1, 2, and 4 in each condition

	Mean of posterior distribution (95% CoI)	Posterior probability of d’ > 1	Posterior probability of d’ > 2	Posterior probability of d’ > 4
Easiest (S0-S100)	3.45 (2.48; 4.43)	> 0.99	> 0.99	0.14
Medium (S20-S100)	0.78 (-0.19^¶^– 1.79)	0.33	0.01	< 0.01
Hardest (S60-S100)	0.16 (-0.81^¶^– 1.17)	0.05	< 0.01	< 0.01

Note: Negative values here imply that the false alarm rate would be higher than the hit rate, meaning that participants were performing very poorly in the task. In practice where this occurred in the present data (6 instances, out of 48 *d’* values that were calculated) *d’* = 0 was assigned

### fMRI data

**Fig. 3:**
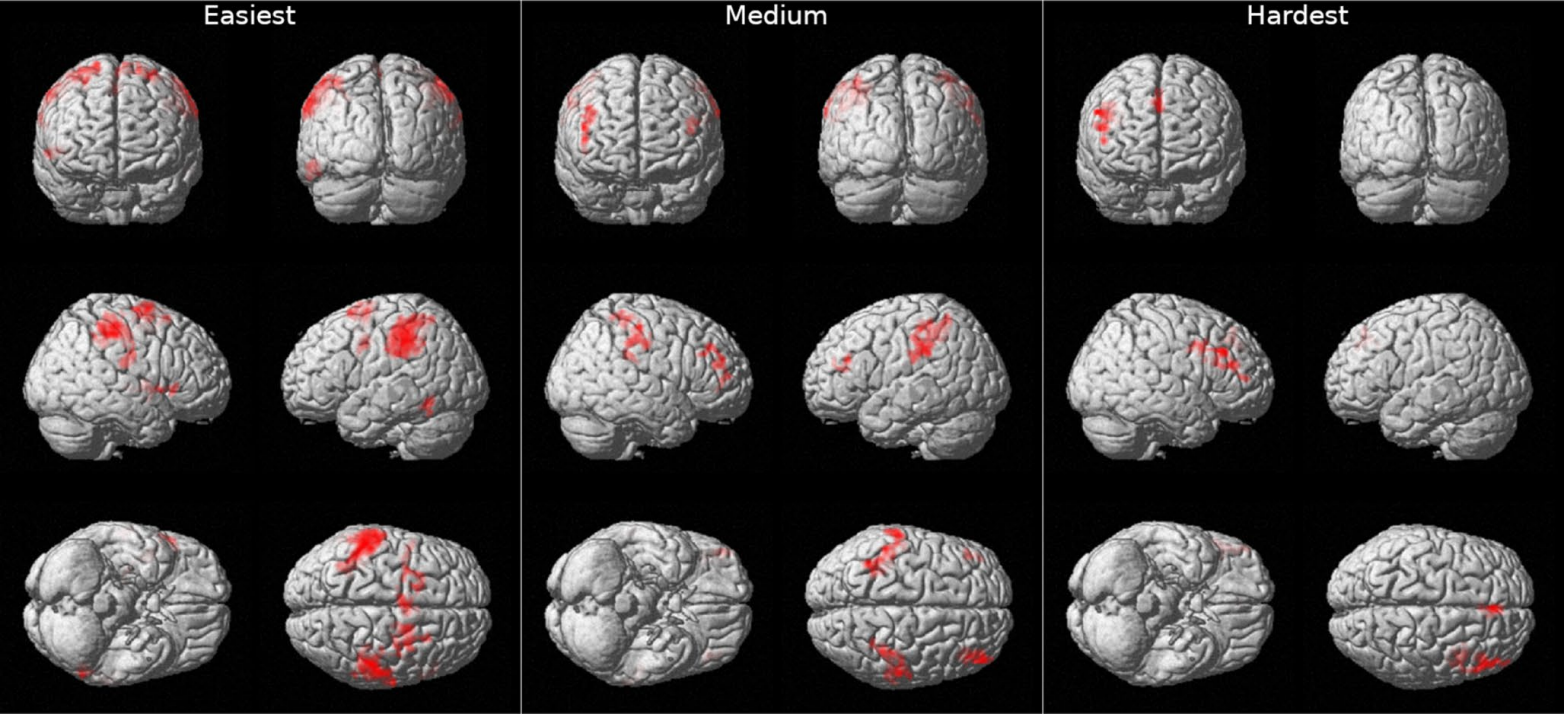
Brain activation for three tactile perceptual discrimination contrasts from the least to the most fine-grained discrimination: Easiest (to the left), Medium (in the middle), Hardest (to the right). Note: For each respective contrast, the top images show the brain from an anterior view (left panel) and a posterior view (right panel), the middle images display lateral views of the right side (left panel) and left side (right panel), and the bottom images illustrate ventral (left panel) and dorsal (right panel) views of the brain

The results of the General Linear Model (GLM) analysis on the brain imaging data indicate that signal location and strength do indeed differ depending on the pairwise comparison being made, see Table 2; Fig. 3. For the “S0-100 vs. S100-S100” contrast (the easiest condition) there were significant activation clusters in the left and right parietal lobe (postcentral, supramarginal, inferior parietal), insular cortex, the temporal lobe (inferior temporal) and the frontal lobe (superior frontal). For the “S20-100 vs. S100-S100” contrast (the medium difficult condition) there were significant activation clusters in the left parietal lobe (inferior parietal, supramarginal, postcentral), right parietal lobe (supramarginal, postcentral), the right frontal lobe (middle frontal, inferior frontal gyrus, triangularis) and the left frontal lobe (middle frontal, inferior frontal gyrus, triangularis). For the “S60-100 vs. S100-100” contrast (the most difficult condition), there were activation clusters in the right frontal lobe (middle frontal, inferior frontal, precentral, superior medial). Thus, as task difficulty increases, activation shifts the findings reveal a dynamic shift in brain activation patterns, from bilateral parietal regions in the easiest discrimination task to predominantly frontal activation, particularly in the right hemisphere, as the difficulty of tactile perceptual discrimination tasks increases.

**Table 2:** Activated brain clusters in each tactile discrimination contrast from the general linear model analysis

Brain region	Hemisphere	Cluster size (voxels)	MNI coordinates (peak voxel)			T-value	Z-value
			x	y	z		
**Easiest (S0-S100 vs. S100-S100)** *cluster size > 147*							
Postcentral, SupraMarginal, Parietal_Inf	R	1138	48	-30	46	7.73	4.75
Postcentral, SupraMarginal, Parietal_Inf	L	1650	-46	-44	64	7.13	4.56
Insula	R	335	42	-4	0	5.82	4.08
Temporal_Inf	L	159	-56	-54	-12	5.60	3.99
Precentral	L	147	-42	-4	42	5.56	3.98
Sup_Motor_Area	L	330	-6	-4	68	5.52	3.96
Frontal_Sup	R	469	18	4	74	5.51	3.96
Frontal_Sup	L	241	-24	12	62	4.98	3.72
**Medium (S20-S100 vs. S100-S100)** *cluster size > 104*							
Parietal_Inf, SupraMarginal, Postcentral	L	996	-40	-42	48	9.65	5.26
SupraMarginal, Postcentral	R	609	56	-28	44	7.27	4.61
Frontal_Mid, Frontal_Inf_Tri	R	268	48	40	20	6.79	4.45
Frontal_Mid, Frontal_Inf_Tri	L	104	-48	32	24	5.23	3.83
**Hardest (S60-S100 vs. S10100)** *cluster size > 236*							
Frontal_Mid, Frontal_Inf_Tri, Precentral	R	543	50	34	20	6.98	4.51
Frontal_Sup_Mid	R	236	8	34	42	5.81	4.08

Note: (*p* < 0.001 uncorrected) Hemisphere (R = right, L = left), T indicates peak t-values, Z indicates peak z-values, Sup = superior gyrus, Inf = inferior gyrus, Mid = middle gyrus, Tri = triangular part

## Discussion

The primary objective of our study was to map the neural circuits involved in a tactile perceptual discrimination task, where participants distinguished surfaces with varying topographical and frictional properties across increasing levels of difficulty. By using fMRI, we aimed to capture the cerebral activations associated with these tactile judgments. Our findings, which corroborate previous behavioral tactile research (Skedung et al. 2018b), reveal differential brain activation patterns contingent on the difficulty of the discrimination task. Thus, as task difficulty increases, there is a dynamic shift in brain activation patterns—from bilateral parietal regions during the easiest discrimination tasks to predominantly frontal activation, particularly in the right hemisphere, as the difficulty of tactile perceptual discrimination tasks increases. This progression demonstrates the intricate neural processes involved in processing tactile information of varying difficulty levels. Following sections delves into the implications of these findings and proposes avenues for forthcoming inquiries.

In the least difficult condition (S0-100 vs. S100-S100) significant activations were observed bilaterally in the parietal lobes, insular cortex, temporal lobes, and frontal lobes. This widespread activation suggests that even easy tactile tasks engage multiple brain regions for basic sensory processing and integration (Yeon et al. 2017). As the task difficulty increases, the activation pattern shifts. The medium difficult condition (S20-100 vs. S100-S100) shows significant activations in both left and right parietal lobes, as well as bilateral frontal lobe involvement. This pattern indicates increased engagement of higher-order cognitive processes as the discrimination task becomes more challenging (Andres et al. 2012), such as differing demands on working memory or attentional resource allocation, factors that future studies should be designed to investigate. In the hardest condition (S60-100 vs. S100-100), activation clusters are predominantly observed in the right frontal lobe. This shift towards frontal dominance, especially in the right hemisphere, suggests a greater reliance on executive functions and higher-order cognitive processes for solving more difficult tactile discrimination tasks (Yeon et al. 2017; Tang et al. 2022).

The observed progression from bilateral parietal activation to right frontal dominance as task difficulty increases aligns with current understanding of tactile perception and cognitive processing. The parietal lobes, particularly the postcentral gyrus (primary somatosensory cortex), play a crucial role in initial tactile information processing (Tang et al. 2022). Their bilateral activation in easier tasks reflects basic sensory processing and integration. As task difficulty increases, there’s greater involvement of frontal regions, particularly the middle and inferior frontal gyri. This suggests increased engagement of working memory, attention, and decision-making processes necessary for more challenging discriminations (Andres et al. 2012). The shift towards right frontal dominance in the hardest condition may indicate specialized processing for difficult spatial and tactile tasks (Sadato et al. 2000). This aligns with the right hemisphere’s known role in spatial processing and attention (Shulman et al. 2010). The progression from parietal to frontal activation suggests a shift in processing demands, where simpler, less challenging sensory discriminations rely more on parietal regions associated with basic perceptual processing, while more complex, demanding discriminations engage frontal areas involved in higher-order cognitive functions such as decision-making, attention, and working memory (Yeon et al. 2017; Tang et al. 2022). This study provides valuable insights into the neural mechanisms underlying tactile perceptual discrimination of varying difficulty, highlighting the brain’s adaptive capacity to engage different regions as task difficulty increase in line with research in other domains such as visual perception (Ide et al. 2016), working memory (Flegal et al. 2019) and cognitive control (Atiani et al. 2009). These examples across various cognitive domains highlight a common principle: as task difficulty increases, the brain adapts by recruiting additional neural resources to meet the increased cognitive demands. This adaptive capacity allows for flexible and efficient processing across a wide range of task complexities.

In interpreting our results, one important factor to consider is the potential role of motor and sensory imagery during the 4-second response period. Motor imagery and tactile discrimination play crucial roles in shaping neural activation patterns during complex sensorimotor tasks. Recent studies have highlighted the importance of considering these factors when interpreting brain activation data (e.g. Debarnot et el., 2021: Meugnot et al. 2015). This highlights the complex interplay between motor imagery, tactile discrimination, and neural activation patterns. Future research should consider these factors when designing experiments and interpreting results in sensorimotor studies. Other important factors to consider are, for example, memory and attention, so future studies should also investigate these factors in the context of tactile discrimination and brain function.

The current study has some potential limitations. For example, the manual presentation of tactile stimuli may have introduced variability in factors such as duration and delivery angles. A more controlled, mechanical method of stimulus presentation could reduce such variability. However, the human-to-human nature of stimulus delivery may also have positively influenced participants’ tactile discrimination abilities by enhancing attention, engagement, or motivation. In addition, the interpersonal interaction might have activated social and emotional cognitive processes that could shape tactile perception in ways that a purely mechanical setup would not. Future research could delve deeper into the potential social and psychological effects of human-mediated tactile presentation to better understand its impact on tactile processing. Future studies should also aim to address other limitations, such as expanding the participant pool to ensure broader generalizability and increased statistical power. Including older adults will be particularly crucial to determine whether and how the findings apply across the human lifespan, as aging has been shown to significantly affect tactile perception (Skedung et al. 2018b). Given that only women were included in the present study, and that previous research has shown that men and women can differ in their tactile sensation (Sadato et al. 2000; Peters et al. 2009), this represents another factor that limits the generalizability of our findings. Additionally, investigating other types of tactile stimulus discrimination, such as stickiness or texture, will help clarify whether the observed patterns are generalizable to all tactile stimuli or specific to roughness discrimination.

To deepen our understanding of tactile perception discrimination and its neural mechanisms, future research should consider adopting single-subject methodologies (Fischer et al. 2023) alongside group-based approaches. Further exploration of the interplay between cognitive, sensory, and motor mechanisms in tactile perception using single-subject approaches could provide deeper insights into the complexities of tactile information processing in the human brain.

In conclusion, this study’s findings shed light on the diverse neural mechanisms underpinning tactile perceptual discrimination, emphasizing the complexity of tactile decision-making. These results contribute to understanding and addressing the decline in tactile acuity, particularly in the elderly and in individuals suffering from long COVID, where 60% of long-term COVID patients exhibit reduced tactile ability (Graham et al. 2021). It is also well-documented that tactile discrimination ability declines with age (Skedung et al. 2018b), making this an important area for further exploration. The methodology and findings from this study have broader implications, particularly for developing strategies related to “tactile communication”, such as safety applications, assisting the visually impaired, and even marketing.

## Electronic supplementary material

Below is the link to the electronic supplementary material.

Supplementary Material 1

## Data Availability

The data supporting the conclusions of this article will be made available by the authors upon reasonable request.

## References

[CR1] Ackerley R, Hassan E, Curran A, Wessberg J, Olausson H, McGlone F (2012) An fMRI study on cortical responses during active self-touch and passive touch from others. Front Behav Neurosci 6:51. 10.3389/fnbeh.2012.00051 22891054 10.3389/fnbeh.2012.00051 PMC3412995

[CR2] Andres M, Pelgrims B, Olivier E (2012) Distinct contribution of the parietal and Temporal cortex to hand configuration and contextual judgments about tools. Cortex 49:2097–2108. 10.1016/j.cortex.2012.11.013 23313011 10.1016/j.cortex.2012.11.013

[CR3] Arvidsson M, Ringstad L, Skedung L, Duvefelt K, Rutland MW (2017) Feeling fine: the effect of topography and friction on perceived roughness and slipperiness. Biotribology 11:92–101. 10.1016/J.BIOTRI.2017.01.002

[CR4] Ashburner J, Friston KJ (2005) Unified segmentation. NeuroImage 26:839–851. 10.1016/j.neuroimage.2005.02.018 15955494 10.1016/j.neuroimage.2005.02.018

[CR5] Atiani S, Elhilali M, David SV, Fritz JB, Shamma SA (2009) Task difficulty and performance induce diverse adaptive patterns in gain and shape of primary auditory cortical receptive fields. Neuron 61:467–480. 10.1016/j.neuron.2008.12.027 19217382 10.1016/j.neuron.2008.12.027 PMC3882691

[CR6] Bendtsen M (2018) A gentle introduction to the comparison between null hypothesis testing and bayesian analysis: reanalysis of two randomized controlled trials. J Med Internet Res 20:e10873. 10.2196/10873 30148453 10.2196/10873 PMC6231868

[CR7] Bergmann Tiest WM, Kahrimanovic M, Niemantsverdriet I, Bogale K, Kappers AML (2012) Salient material properties and haptic volume perception: the influences of surface texture, thermal conductivity, and compliance. Atten Percept Psychophys 74:1810–1818. 10.3758/s13414-012-0372-2 22972632 10.3758/s13414-012-0372-2

[CR8] Bolanowski SJ, Verrillo RT, McGlone F (1999) Passive, active and intra-active (self) touch. Somatosens Mot Res 16:304–311. 10.1080/08990229970375 10632028 10.1080/08990229970375

[CR9] Carpenter CW, Dhong C, Root NB, Rodriquez D, Abdo EE, Skelil K, Alkhadra MA, Ramirez J, Ramachandran VS, Lipomi DJ (2018) Human ability to discriminate surface chemistry by touch. Mater Horiz 5:70–77. 10.1039/C7MH00800G

[CR10] Collier ES, Lawson R (2016) Defining filled and empty space: reassessing the filled space illusion for active touch and vision. Exp Brain Res 234:2697–2708. 10.1007/s00221-016-4673-x 27233286 10.1007/s00221-016-4673-x PMC4978768

[CR11] Debarnot U, Perrault AA, Sterpenich V, Legrand N, Huber C, Moisseev A, Hubert D, Schwartz S, Guillot A (2021) Motor imagery practice benefits during arm immobilization. Sci Rep 11:8928. 10.1038/s41598-021-88142-6 33903619 10.1038/s41598-021-88142-6 PMC8076317

[CR12] Fischer H, Nilsson ME, Ebner NC (2023) Why the single-N design should be the default in affective neuroscience. Affect Sci. 10.1007/s42761-023-00182-5 38495781 10.1007/s42761-023-00182-5 PMC10942943

[CR13] Flegal KE, Ragland JD, Ranganath C (2019) Adaptive task difficulty influences neural plasticity and transfer of training. NeuroImage 188:111–121. 10.1016/j.neuroimage.2018.12.003 30521951 10.1016/j.neuroimage.2018.12.003 PMC6401296

[CR14] Fleming MS, Luo W (2013) The anatomy, function, and development of mammalian Aβ low-threshold mechanoreceptors. Front Biol 8:408–420. 10.1007/s11515-013-1271-1 10.1007/s11515-013-1271-1 PMC3873732 24376457

[CR15] Graham EL, Clark JR, Orban ZS, Lim PH, Szymanski AL, Taylor C, DiBiase RM, Jia DT, Balabanov R, Ho SU, Batra A, Liotta EM, Koralnik IJ (2021) Persistent neurologic symptoms and cognitive dysfunction in non-hospitalized COVID-19 long haulers. Ann Clin Transl Neurol 8:1073–1085. 10.1002/acn3.51350 33755344 10.1002/acn3.51350 PMC8108421

[CR16] Gueorguiev D, Bochereau S, Mouraux A, Hayward V, Thonnard JL (2016) Touch uses frictional cues to discriminate flat materials. Sci Rep 6:25553. 10.1038/srep25553 27149921 10.1038/srep25553 PMC4858763

[CR17] Harris JA, Harris IM, Diamond ME (2001) The topography of tactile learning in humans. J Neurosci 21:1056–1061. 10.1523/JNEUROSCI.21-03-01056.2001 11157091 10.1523/JNEUROSCI.21-03-01056.2001 PMC6762328

[CR18] Henderson J, Mari T, Hewitt D, Newton-Fenner A, Giesbrecht T, Marshall A, Stancak A, Fallon N (2023) The neural correlates of texture perception: A systematic review and activation likelihood Estimation meta-analysis of functional magnetic resonance imaging studies. Brain Behav 13:e3264. 10.1002/brb3.3264 37749852 10.1002/brb3.3264 PMC10636420

[CR19] Hua-Chun S, Welchman AE, Chang DHF, Di Luca M (2016) Look but don’t touch: visual cues to surface structure drive somatosensory cortex. NeuroImage 128:353–361. 10.1016/j.neuroimage.2015.12.054 26778128 10.1016/j.neuroimage.2015.12.054 PMC4767223

[CR20] Ide M, Hidaka S, Ikeda H, Shinohara M (2016) Neural mechanisms underlying touch-induced visual perceptual suppression: an fMRI study. Sci Rep 6:37301. 10.1038/srep37301 27874038 10.1038/srep37301 PMC5118811

[CR24] Johnson KO (2001) The roles and functions of cutaneous mechanoreceptors. Curr Opin Neurobiol 11:455–461. 10.1016/S0959-4388(00)00234-8 11502392 10.1016/s0959-4388(00)00234-8

[CR21] Katz D (1925) Der aufbau der tastwelt. Ergaenzungsband 11, zeitschrift für psychologie und physiologie der Sinnesorgane. Johann Ambrosius Barth

[CR22] Katz D (1989) The world of touch. Hillsdale, Erlbaum

[CR23] Kuroki S, Watanabe J, Nishida SY (2017) Integration of vibrotactile frequency information beyond the mechanoreceptor channel and somatotopy. Sci Rep 7:2758. 10.1038/s41598-017-02922-7 28584282 10.1038/s41598-017-02922-7 PMC5459808

[CR25] Lederman S, Gati J, Servos P, Wilson D (2001) fMRI-derived cortical maps for haptic shape, texture, and hardness. Cogn Brain Res 12:307–313. 10.1016/S0926-6410(01)00041-6 10.1016/s0926-6410(01)00041-6 11587899

[CR26] McElreath R (2018) Statistical rethinking: A bayesian course with examples in R and Stan. Chapman Hall/CRC. 10.1201/9781315372495

[CR27] McGlone F, Wessberg J, Olausson H (2014) Discriminative and affective touch: sensing and feeling. Neuron 82:737–755. 10.1016/j.neuron.2014.05.001 24853935 10.1016/j.neuron.2014.05.001

[CR28] Meugnot A, Agbangla NF, Almecija Y, Toussaint L (2015) Motor imagery practice May compensate for the slowdown of sensorimotor processes induced by short-term upper-limb immobilization. Psychol Res 79:489–499. 10.1007/s00426-014-0577-1 24908074 10.1007/s00426-014-0577-1

[CR29] Okamoto S, Nagano H, Yamada Y (2012) Psychophysical dimensions of tactile perception of textures. IEEE Trans Haptics 6:81–93. 10.1109/TOH.2012.32 10.1109/TOH.2012.32 24808270

[CR30] Olausson H, Lamarre Y, Backlund H, Morin C, Wallin BG, Starck G, Ekholm S, Strigo I, Worsley K, Vallbo AB, Bushnell MC (2002) Unmyelinated tactile afferents signal touch and project to insular cortex. Nat Neurosci 5:900–904. 10.1038/nn896 12145636 10.1038/nn896

[CR31] Peters RM, Hackeman E, Goldreich D (2009) Diminutive digits discern delicate details: fingertip size and the sex difference in tactile Spatial acuity. J Neurosci 29:15756–15761. 10.1523/JNEUROSCI.3684-09.2009 20016091 10.1523/JNEUROSCI.3684-09.2009 PMC3849661

[CR32] Sadato N, Ibañez V, Deiber MP, Hallett M (2000) Gender difference in premotor activity during active tactile discrimination. NeuroImage 11:532–540. 10.1006/nimg.2000.0566 10806038 10.1006/nimg.2000.0566

[CR33] Shulman GL, Pope DL, Astafiev SV, McAvoy MP, Snyder AZ, Corbetta M (2010) Right hemisphere dominance during Spatial selective attention and target detection occurs outside the dorsal frontoparietal network. J Neurosci 30:3640–3651. 10.1523/JNEUROSCI.4085-09.2010 20219998 10.1523/JNEUROSCI.4085-09.2010 PMC2872555

[CR34] Simões-Franklin C, Whitaker TA, Newell FN (2011) Active and passive touch differentially activate somatosensory cortex in texture perception. Hum Brain Mapp 32:1067–1080. 10.1002/hbm.21091 20669167 10.1002/hbm.21091 PMC6870332

[CR35] Skedung L, Arvidsson M, Chung JY, Stafford CM, Berglund B, Rutland MW (2013) Feeling small: exploring the tactile perception limits. Sci Rep 3:2617. 10.1038/srep02617 24030568 10.1038/srep02617 PMC3771396

[CR36] Skedung L, Harris K, Collier ES, Arvidsson M, Wäckerlin A, Haag W, Bieri M, Romanyuk A, Rutland MW (2018a) Feeling smooth: psychotribological probing of molecular composition. Tribol Lett 66:138. 10.1007/s11249-018-1077-z

[CR37] Skedung L, El Rawadi C, Arvidsson M, Farcet C, Luengo GS, Breton L, Rutland MW (2018b) Mechanisms of tactile sensory deterioration amongst the elderly. Sci Rep 8:5303. 10.1038/s41598-018-23688-6 29674633 10.1038/s41598-018-23688-6 PMC5908919

[CR38] Skedung L, Harris KL, Collier ES, Rutland MW (2020) The finishing touches: the role of friction and roughness in haptic perception of surface coatings. Exp Brain Res 238:1511–1524. 10.1007/s00221-020-05831-w 32447410 10.1007/s00221-020-05831-w PMC7286865

[CR39] Tang Y, Wang F, Hao Y, Liu Y, Tao J (2022) Study of event-related potentials by withdrawal friction on the fingertip. Skin Res Technol 28:320–328. 10.1111/srt.13232 10.1111/srt.13232 PMC9838764 36428289

[CR40] Yeon J, Kim J, Ryu J, Park JY, Chung SC, Kim SP (2017) Human brain activity related to the tactile perception of stickiness. Front Hum Neurosci 11:8. 10.3389/fnhum.2017.00008 28163677 10.3389/fnhum.2017.00008 PMC5247468

