# Supplementary material for: Active touch in tactile perceptual discrimination: brain activity and behavioral responses to surface differences
Source: Exp Brain Res. 2025 Mar 6;243(4):84. doi: 10.1007/s00221-025-07034-7 (PMC11885392; doi:10.1007/s00221-025-07034-7)
Supplement: Supplementary file 1 — Supplementary Material 1 [file 221_2025_7034_MOESM1_ESM.docx]

**Supplementary material**

**Figure 1.** Example of a Design Matrix from One of the First-Level Contrasts (S0-S100 vs. S100-S100).


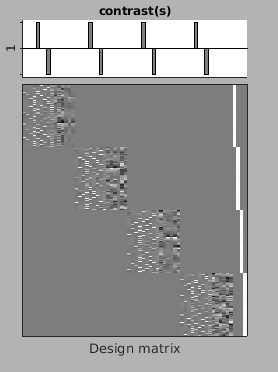


The topography of the wrinkled surfaces, visualized in Figure 2, was characterized with a stylus profilometer (DektakXT Profiler, Nano GmbH, Germany). Area images (1x1 cm) were acquired by moving the stylus tip (radius 2 μm) across the wrinkles in 250 line scans (1.1 mm). The force of the stylus on the surface was set to 3 mg. Data analyses were made in the Vision64 software program. The wavelengths of the surfaces were obtained from the stylus analysis and the parameter PSm - the average peak spacing. The same wavelengths are measured on surfaces that have been used in repeated testing, confirming that the wrinkles are robust towards repeated touching.

**
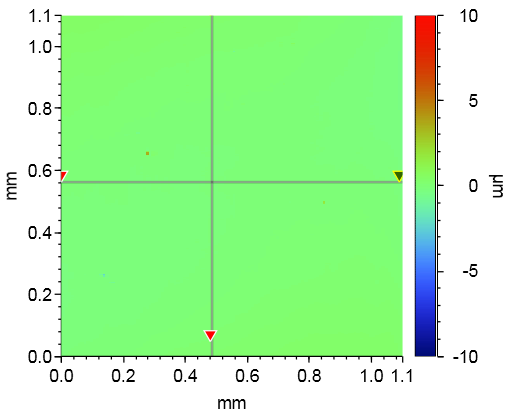

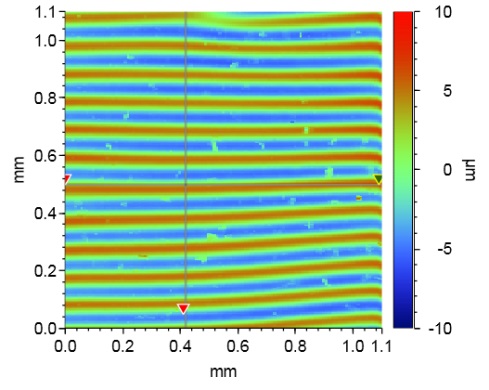

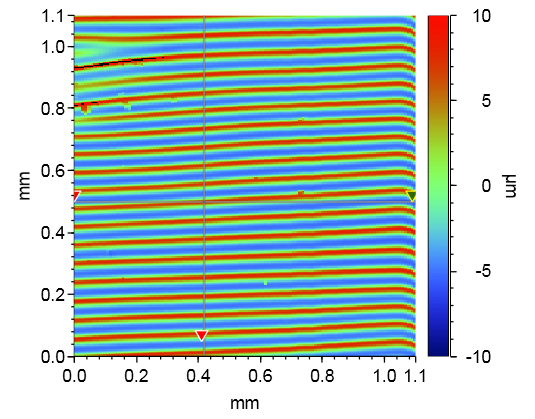

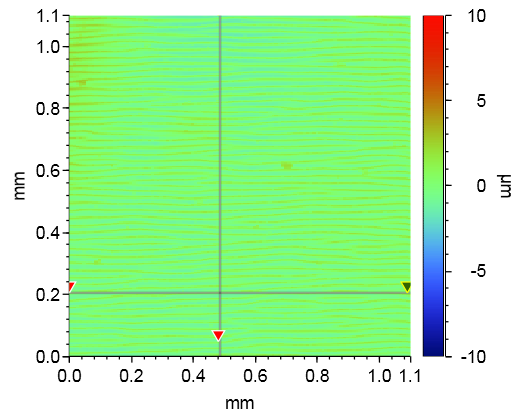

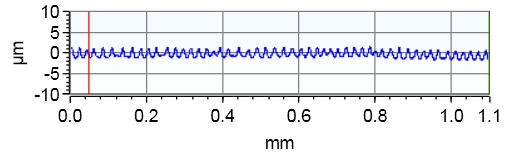

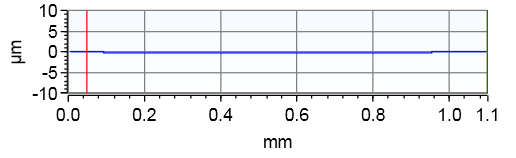

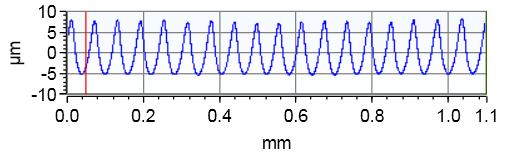

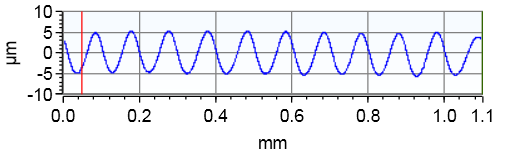
Figure 2.** The topography of the wrinkled surfaces.

S0

S20

S60

S100
